# Toxicity assessment following conventional radiation therapy and pulsed low dose rate radiation therapy: an in vivo animal study

**DOI:** 10.1186/s13014-024-02545-z

**Published:** 2024-11-13

**Authors:** Noha Roshdy Salem, Ahmed Eldib, E. M. El-Sayed, Ehab Mostafa, Omar S. Desouky

**Affiliations:** 1 Radiation oncology Department, Faculty of Medicine, Ain Shams University, Cairo, Egypt; 2 Radiation Oncology Department, Fox Chase Cancer Center, Philadelphia, PA USA; 3 Physics Department, Faculty of Science, Ain Shams University, Cairo, Egypt; 4 Radiation Physics Department, Egyptian Atomic Energy Authority (EAEA), Cairo, Egypt

**Keywords:** Pulsed low dose rate, Conventional radiotherapy, TGF-β, Comet assay, IRS

## Abstract

**Background:**

Pulsed low dose rate radiotherapy (PLDR) is a new radiation delivery method, in which the fractional dose is divided into sub-fractional doses with periodical time breaks in between. The goal of our study is to assess the toxicity on healthy tissues resulting from PLDR as compared to conventional radiotherapy (CRT) using the same physical X-ray dose.

**Methods:**

We analyzed the weight and survival time for CRT and PLDR groups and studied the inflammatory cytokine transforming Growth Factor-β (TGF-β), usually released following irradiation. Histopathological and immunohistochemical analyses were conducted for intestinal and bone marrow tissues from rats subjected to 8 Gy whole- body irradiation using CRT and PLDR techniques. We investigated genotoxicity by performing a comet assay (CA) in splenic tissues.

**Results:**

Our findings showed an improvement in survival time with PLDR versus CRT by 82%.The mean survival time for CRT rats’ group was 6.3 days, while it was 35.9 days for PLDR group.The weight of CRT group decreased gradually by 3.7%, while weight of PLDR group increased gradually by 2.4%.CRT resulted in more cellular atrophy in bone marrow and intestinal tissues than in PLDR treatments as shown by hematoxylin and eosin staining analysis. In addition, the transforming growth factor-β (TGF-β) expression in bone marrow and intestinal tissues of CRT was higher than those expressed in tissues from PLDR as demonstrated by the Immuno reactive score (IRS). It was10(0.53) and 9.8(0.55) for BM and intestinal tissues, respectively from CRT group and 5.8(0.63) for PLDR for both tissues. The measured CA parameters were larger with CRT compared to PLDR, where the Tail Length (TL), Tail DNA % (TD%) and Tail Moment (TM) measurements were 25.4(3.4), 56.5(7.6) % and 20.5(3.5) for CRT, 7.3(1.9), 30.0(7.2) % and 5.7(1.8) for PLDR, with *P* value 0.000064, 0.0004 and 0.00017, respectively.

**Conclusion:**

This study indicates that PLDR can reduce the toxicity on normal tissues compared to CRT.

**Supplementary Information:**

The online version contains supplementary material available at 10.1186/s13014-024-02545-z.

## Introduction

Pulsed low dose rate radiation therapy is a new radiation delivery method, that has been proposed to overcome the unacceptable expected toxicity when re-irradiating recurrent cancers that have already received prior radiotherapeutic doses [1–4].The basic idea behind PLDR is to take advantage of the low dose hyper-radiosensitivity (HRS) of tumor cells [5, 6] below certain threshold doses, which are greater than those of normal tissues. In addition, the low dose rate used with PLDR will allow for a better healthy tissue repair [7–9]. The potential interpretation for the low dose HRS is the lack of DNA repair below this given threshold dose which is called the transition dose. This is the dose in which cells change from hyper-radiosensitivity to resistance response. This can be noticed in the cells survival curves as a region of increased radioresistance (IRR). This dose depends on cell type and usually determined to be in the range of 0.2–0.6 Gy [10–25]. Thus, contrary to the normal tissue repair of the sub-lethal DNA damage at low radiation dose rates, tumor cells are shown to be more radiosensitive as long as the radiation pulse doesn’t exceed the transition dose. The phenomenon of higher radiosensitivity at lower dose rate is also called the inverse dose rate effect [10–12] and could be seen at radiation dose rates less than 1 Gy/h in low dose HRS-expressing cells [25–27]. Survival curves for invitro studies have shown that the effect of dose rate on sublethal damage is clearly observed in the range of 0.01–1 Gy/h [6]. To achieve this effect in PLDR, the radiation treatment fraction is divided into a number of sub-fractions or pulses, each one has a dose higher than the transition dose for normal tissue but lower than the transition dose for the tumor, this promotes DNA repair in normal tissues, but not in cancerous cells. To maximize normal tissue repair, pulses are delivered with predetermined periodic time interval breaks to achieve an effective low dose rate [3].

PLDR has been investigated through in vitro/in vivo radiobiological experiments [5, 7, 28–35]. Those studies have guided pilot clinical studies for the treatment of recurrent cancers and aided in determining the schemes of fractionation and dose rate for specific tumor sites. For a variety of used tumor cell lines, the majority of PLDR treatments resulted in comparable or somewhat better cell death than CRT. The varied response of the different cell lines probably indicates a limited degree of efficacy in preventing the activation of the early G2 checkpoint and subsequent DNA repair [9]. It is anticipated that the distinction between the in vivo and in vitro environments will also have an impact on the signaling pathways that govern the early G2 checkpoint, which is sustained by multiple important kinases and phosphorylation processes and triggered by ATM (ataxia-telangiectasia mutated) activity [9]. Most in-vitro studies used colony assays to evaluate PLDR- based HRS. In those studies, different cell lines were investigated by assessing the cell survival rates with PLDR versus conventional methods [28–30, 36]. For example, Todorovic et al. [28] used a clonogenic assay to evaluate the response of different isogenic HNSCC Cell lines to PLDR treatment. On the other hand, most in-vivo studies evaluated the PLDR effect by monitoring the tumor growth delay using different imaging modalities. For example, Dilwarth et al. and Park et al. used positron emission tomography (PET) scans to quantify tumor growth of Glioblastoma multiform tumors established in nude mice after irradiation with PLDR compared to conventional fractionation [32, 33]. Zhang et al. used magnetic resonance imaging (MRI) to monitor the weekly growth of lung tumor model implanted in mice to study the effectiveness of PLDR [31]. Wang et al. also used MRI but to investigate the efficiency of PLDR for treating implanted in-vivo prostate cancers [5]. Other factors were also used to evaluate PLDR effect in some in-vivo studies. For example, Meyer et al. analyzed the toxicity by measuring Transforming Growth Factor-β (TGF-β) in tissues from mice that were irradiated with PLDR using lethal doses [35].The in-vivo published studies presented valuable data to aid in the clinical trials, but are still not enough to interpret why clinical toxicity is reduced in PLDR treatment technique and the mechanism of radiation damage repair in the cells is still not fully understood at the molecular level [6, 37]. We believe more research efforts are needed to have a better understanding of this new treatment technique. Therefore, we were motivated to perform in vivo experiments using rats to investigate the different effect of PLDR on normal cells compared to the effect of conventional treatments. PLDR effects could be ascribed to DNA damage repair mechanisms prematurely triggered by the accumulation of sufficient damage from the repeated dose pulses. In our study, we conducted a comet assay, a technique for quantifying and analyzing DNA damage and repair in individual cells. As far as our knowledge, comet assays were not performed in the early studies for evaluating PLDR based on in-vivo experiments. We also measured TGF-β in different tissues because it is considered the main cytokine responsible for the fibrotic response in healthy tissues after exposure to radiation and an indicator of tissue damage [38, 39].

## Materials & methods

### Animals

Adult male Wistar rats were obtained from Medicine Ain Shams Medical Research Institute (MASRI), weigh 130–150 gm and approximately 8 weeks old. They were kept in cages made of polypropylene in conventional laboratory condition (temperature 25 °C ± 2 °C, 50 ± 10% relative humidity, and a 12-h cycle of darkness and light). The animals were fed a regular standard diet, and water was available ad Libitum. Prior to the start of the study, all rats spent a week getting used to the surroundings.

### Experimental design

Our experiments were performed at two stages, the first stage focused on the difference in survival time between PLDR and CRT. The assessment was done using 24 rats divided into two groups, Pulsed low dose rate group and the conventional radiotherapy group. In the second stage, we conducted toxicity and histopathological examinations using 38 rats divided into three groups, 13 rats for the Pulsed low dose rate group, 13 rats for conventional radiotherapy group, and 12 rats for the control group. The comet assay was compared between 6 rats from each group. TGF-β measurements were compared in 7 rats from each group. We used the Wilcoxon Rank Sum test to calculate P-value.

### Irradiation

Rats were placed in a box confining them within an area of 12 cm × 12 cm. An arbitrary reference point was placed within the center of the box to calculate the machine monitor units needed to deliver a dose in the order of 8 Gy ± 0. 8 Gy.Treatment was carried out using parallel opposed lateral beams, covering the rat’s whole body plus some marginal area to ensure that the rat is not standing within the field penumbra. Both CRT and PLDR groups received total body irradiation to the same dose but with different delivery methods. Conventional radiotherapy (CRT) was delivered as a single 8 Gy dose using a machine dose rate equal to 400 MU/minute, whereas PLDR was delivered via 40 × 0.2 Gy pulses. Each pulse is running at 100 MU/minute and pulses are separated by 3 min interval beam off gaps to achieve an effective dose rate equal to 0.067 Gy/min. The rats were irradiated using a Unique linear accelerator (Varian medical systems) witha6 MV photon beam. The splenic tissues were resected 48 h after irradiation for DNA damage detection, while bone marrow and gastrointestinal tissues were resected one week after irradiation for histopathology and immunohistochemistry.

### Comet assay

Single cell gel electrophoresis assay (SCGE) or a Comet assay is a common method for measuring DNA damage in cells described in several publications [40, 41]. This assay detects and quantifies breaks in DNA [42]. The original protocol developed by Singh et al. [40] was followed in this study with some modifications according to the reagents manufacturers. Splenic tissues were extracted, fixed in phosphate puffer saline for processing using the Cell Biolabs, Inc.'s OxiSelectTM 96-Well Comet Assay kit, cat. no. STA-355, San Diego, USA. The test was conducted according to the manufacturer's instructions and the protocol provided in kit handbook [43]. The comet assay method is adapted to determine DNA damage in isolated cells, first, the cells needed to be isolated from splenic tissues. Obtaining a cell suspension of high quality may be tricky when working with tissues. To isolate and lyse splenic tissue, we used dissection scissors to mince a small piece of spleen in 1–2 mL of ice-cold PBS containing 20 mM EDTA (without Mg^2+^ and Ca^2+^). Then we allowed the tissue/cell suspension to stand for5 minutes before transferring the supernatant to a centrifuge tube. the supernatant was discarded, then cells were resuspended at 1 × 10^5^ cells/mL in ice-cold PBS (without Mg^2+^ and Ca^2+^). Before applying the individual cells on the OxiSelectTM 96-Well Comet Slide, the cells were first mixed with molten agarose gel. Then, to relax and denature the DNA, the slide was immersed in Lysis Buffer for 30–60 min at 4 °C in the dark, and then the buffer solution was replaced with alkaline solution for 30 min at 4 °C in the dark. The DNA in these implanted cells was then relaxed and denatured using lysis buffer and alkaline solution. The samples were then electrophoresed in a horizontal chamber to separate the intact DNA from the damaged bits (DNA fragments). The samples were dried, dyed with a DNA dye and examined using epifluorescence microscopy after electrophoresis. Damaged DNA (including strand breaks and cleavages) moved further than intact DNA and generated a "comet tail.". The cells with a comet tail, with damaged DNA, were photographed using LABOMED Fluorescence microscope LX400, cat no: 9126000; USA. and assessed by comet assay analysis software CASPlab. To quantify the extent of the DNA damage the distance between the genetic material in the nucleus (also known as the "comet head") and the subsequent "tail" was measured. Parameters used to evaluate comet assay results were as follows; Tail length (TL) measured from the center of the comet to the tail end, Tail DNA% (TD%) is the DNA percentage in the result tail and Tail Moment (TM) equal to (TD%) multiplied by the Length of Tail (TM = TD% × TL) [44].

### Histopathology and immunohistochemistry

The intestine tissues and bone marrow (BM) were harvested from the different groups (7 rats from each group), placed into cassette, and then immersed in formalin (10% neutral buffered) to be fixed in paraffin blocks for immunohistochemical and histopathological examinations. From these tissues in the various groups, autopsy samples were taken and fixed in 10% formalin saline for 24 h, then washed using tap water (containing ions and cations like calcium, which found to give better results concerning differentiation and color intensity) and dehydration was induced using diluted alcohol (methyl, ethyl, and pure ethyl). In a hot air oven set to 56 °C for 24 h, specimens were immersed in paraffin after cleaning with xylene. The microtome was used to make paraffin bees wax tissue blocks for sectioning at a thickness of 4 microns. The acquired tissue sections were assembled onto glass slides, deparaffinized, and stained using the Hematoxylin and Eosin (H&E) stain to be examined by LABOMED Fluorescence microscope LX400, cat no: 9126000; USA.

### TGF-β

The samples' paraffin wax was removed to carry out antibody staining using xylene. Rehydration started by placing the sections in 100% ethanol followed by 95% ethanol, then washed two times in dH_2_0. Slides were subjected to antigen retrieval by boiling in buffer (10 mM Tris/1 mM EDTA, with pH = 9.0). The activity of endogenous peroxidase was quenched to induce a high background staining using 3% hydrogen peroxide then washed in distilled water (dH_2_O) followed by washing in wash buffer. Large circles were drawn around the samples using a hydrophobic pen, to obtain the highest quality of antibody staining. The sections were blocked for one hour at room temperature using 100–400 μl of the blocking solution then subsequently incubated at the same conditions over night using the diluted primary antibody, rat specific TGF-β polyclonal Antibody, cat no.PA5-85,171, (Invitrogen, ThermoScientific, USA), at 1:500 dilution. On the next day, dilution of the primary antibody was removed, then the sections were cleaned in washing buffer. The immunohistochemically detection kits were allowed, Envision FLEX link Detection Reagent, cat no. K800 (Dako, Denmark) was applied on the samples and incubated for 30 min, then washed. After that, the slides were stained using DAB Chromogen3,3, -diaminobenzidine tetrahydrochloride (Dako, Denmark), then a 100 to 400 μl of SignalStain® DAB (Dako, Denmark) for each section was applied and monitored closely for 1 to10 minutes to allow for a suitable staining intensity. Finally, the sample slides were immersed in dH2O, followed by counter staining with hematoxylin. Then the sections were subjected to two 5-min dH2O washes. The immune staining was evaluated blindly.

### Immunoreactive score calculation

Using a counting grid, the immune-positive cells were enumerated and calculated in each region of interest (ROI). The ROI’s-, stained areas were digitally defined and their percentage was calculated. Immunoreactive score system (IRS) was used to calculate the protein expression intensity for IHC data interpretation. The immunoreactive score system gives a range of 0–12 by multiplying the staining intensity score (0–3) and positive cells proportion score (0–4). The staining intensity arbitrary scale and values for the fraction of positive tumor cells are shown in Table 1 [45].

**Table 1:** The Immunoreactivity scoring system (IRS)

A (percentage of positive cells)	B (intensity of staining)	IRS score (multiplication of A and B) IRS score (A × B): 0–12
0 = no positive cells	0 = no colour reaction (No staining)	0–1 = negative
1 = < 10% of positive cells	1 = mild reaction (Weak staining)	2–3 = mild (weak)
2 = 11–50% positive cells	2 = moderate reaction (Moderate staining)	4–8 = moderate
3 = 51–80% positive cells	3 = intense reaction (Strong staining)	9–12 = strongly positive
4 = > 80% positive cells		

### Data analysis

Microsoft Excel (365) and Excel Stat (XLStat) were used to analyze the data. To illustrate the uncertainty in the weight of the rats, we calculated the standard deviation (SD) and added a linear fitting to them using Origin 2024b. For the other data, the mean and standard error of the mean (SEM) were calculated. The SEM were estimated as the standard deviation divided by the square root of the sample number. Minitab 19 was used to calculate the Wilcoxon Rank Sum Test, which was utilized in statistical analysis. The threshold for a significant difference is *P* value ≤ 0.05. Prism was used to create box and whisker plots, displaying individual points. The Kaplan–Meier survival analysis was used to plot the survival distribution function. The significant difference level is referred to as *p ≤ 0.05, **p ≤ 0.005, ***p ≤ 0.0001.

## Results

### Weight and survival time

**Fig. 1:**
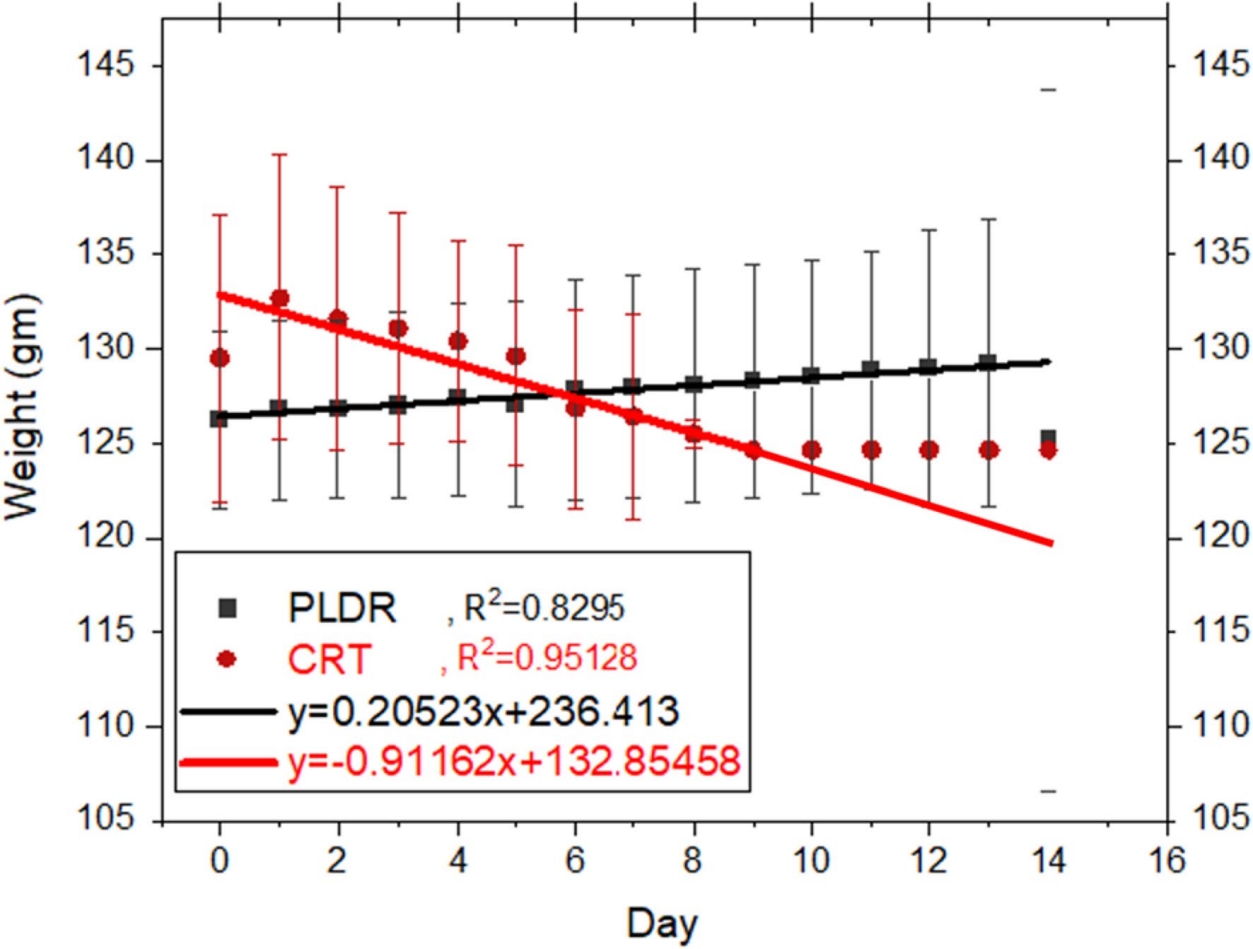
The average weight per day for both rat groups with a linear fitting. CRT group received single 8 Gy and PLDR received Pulsed 8 Gy (0.2 Gy × 40fr with interval time 3 min). Day 0 indicates to time before irradiation

The average weight of the rats in CRT group decreased gradually after the single delivery of the 8 Gy dose. The rats started to die at the third day until all rats died by the 17th day after radiation, while in the PLDR group, the rats’ weight did not decrease (Fig. 1) until the 14th day (PLDR’ rats started to die on 14th day). A significant difference was observed in the initial and final weight for the rats between the two groups with **a** *P* value = 0.001. The average weight before irradiation was 129.5gm and126.2 gm for CRT group and PLDR group, respectively.CRT group’ weight decreased gradually by 3.7% (this decreasing in average weight was from a rapidly decreasing number of surviving rats)) while PLDR group’ weight increased gradually by 2.4%. The highest difference between the weights of the two groups was seen in day 13.

**Fig. 2:**
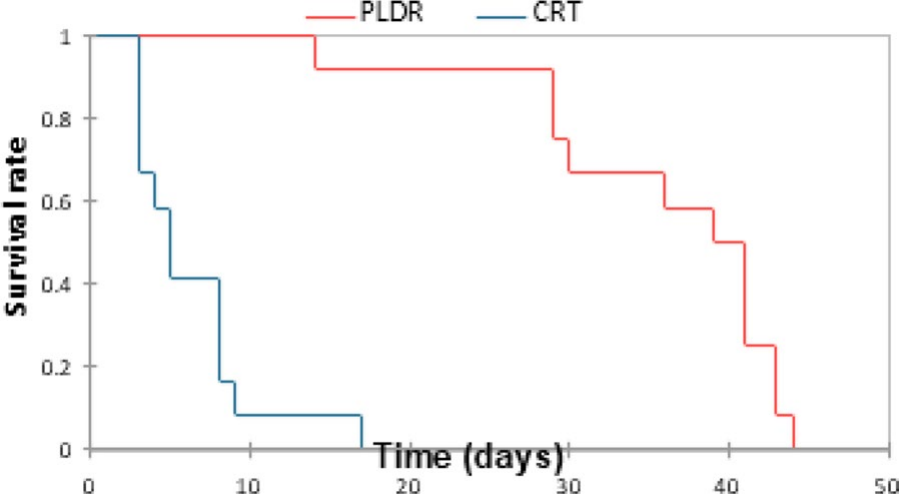
The survival rate using Kaplan Meier plotting. The CRT group received a single 8 Gy dose and the PLDR group received Pulsed 8 Gy (0.2 Gy × 40fractions with interval time 3 min). Day 0 indicates the day before irradiation

Also, a significant difference was observed in the survival rate between the rats in both groups (CRT & PLDR) with a *P* value = 0.001. Kaplan Meier analysis in (Fig. 2) shows that the mean survival time for CRT group is 6.3 days and all rats died by 17th day, while for the PLDR group, the mean survival time is 35.9 days and all rats died by the 44th day. This shows that the survival time was less by 82%with CRT compared to PLDR group. This indicates that PLDR could reduce the toxicity after irradiation as demonstrated with the slower weight decline and longer survival time.

### Symptoms

**Fig. 3:**
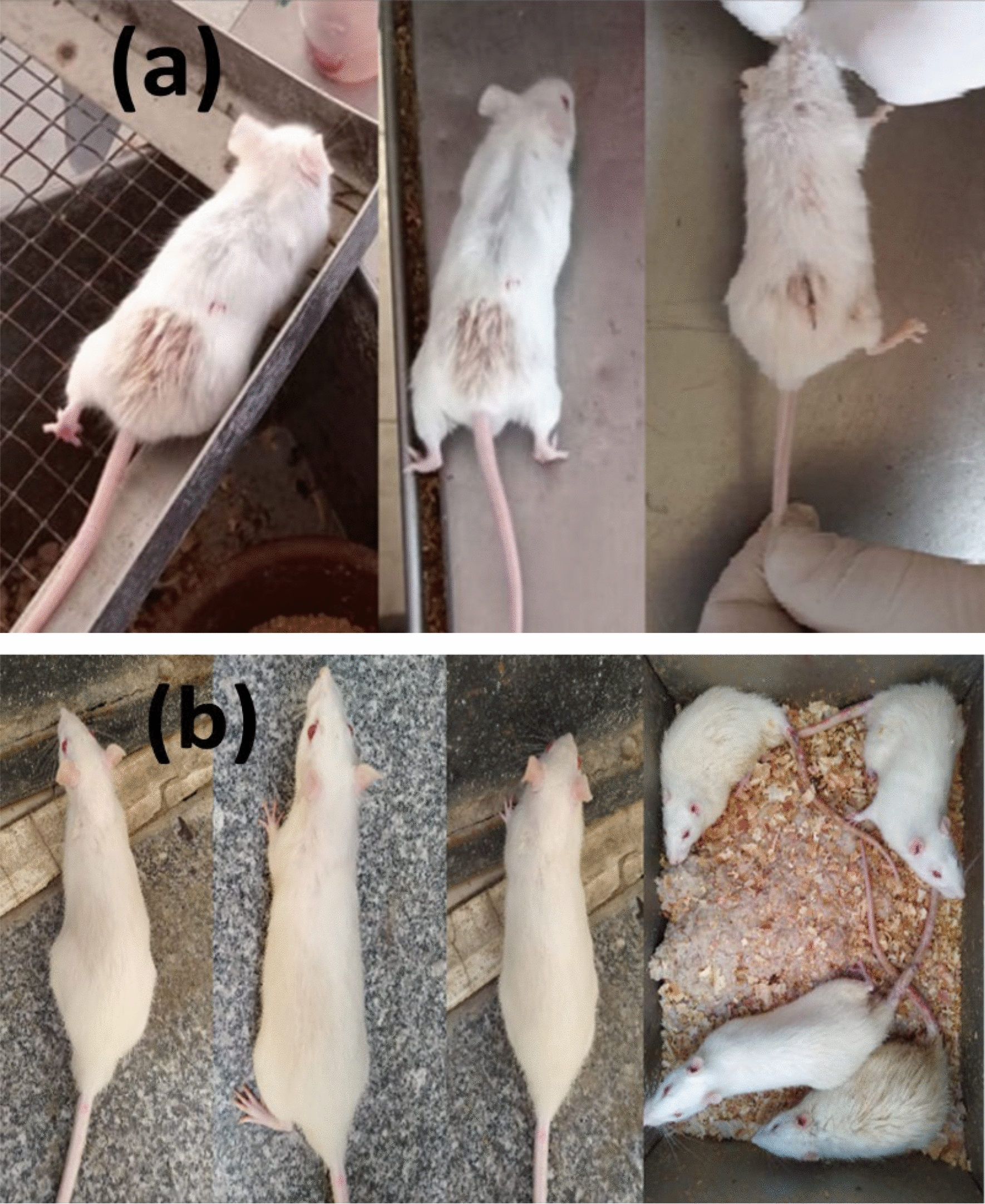
**a** CRT group one week after irradiation; rats which received single8 Gy, showing changes on the skin. **b** PLDR group rats which received pulsed 8 Gy (0.2 Gy × 40fr with interval 3 min), showing normal skin appearance

Acute symptoms of fatigue, lethargy and tremor appeared in the rats after exposure to 8 Gy with conventional radiation delivery. One week after irradiation, scars and erosion in the fur were noticed in some of the rats in the CRT group (Fig. 3a), while rats of the other group showed normal appearance (Fig. 3b). During dissection, gastric dilation was noticed in some rats of both CRT and PLDR groups, but the macroscopic morphologic alterations were higher in the CRT group. The gasified water contributed to the expansion of the gastric area and led to the elimination of the linear depression of the mucous organ following irradiation.

### Comet assay

**Fig. 4:**
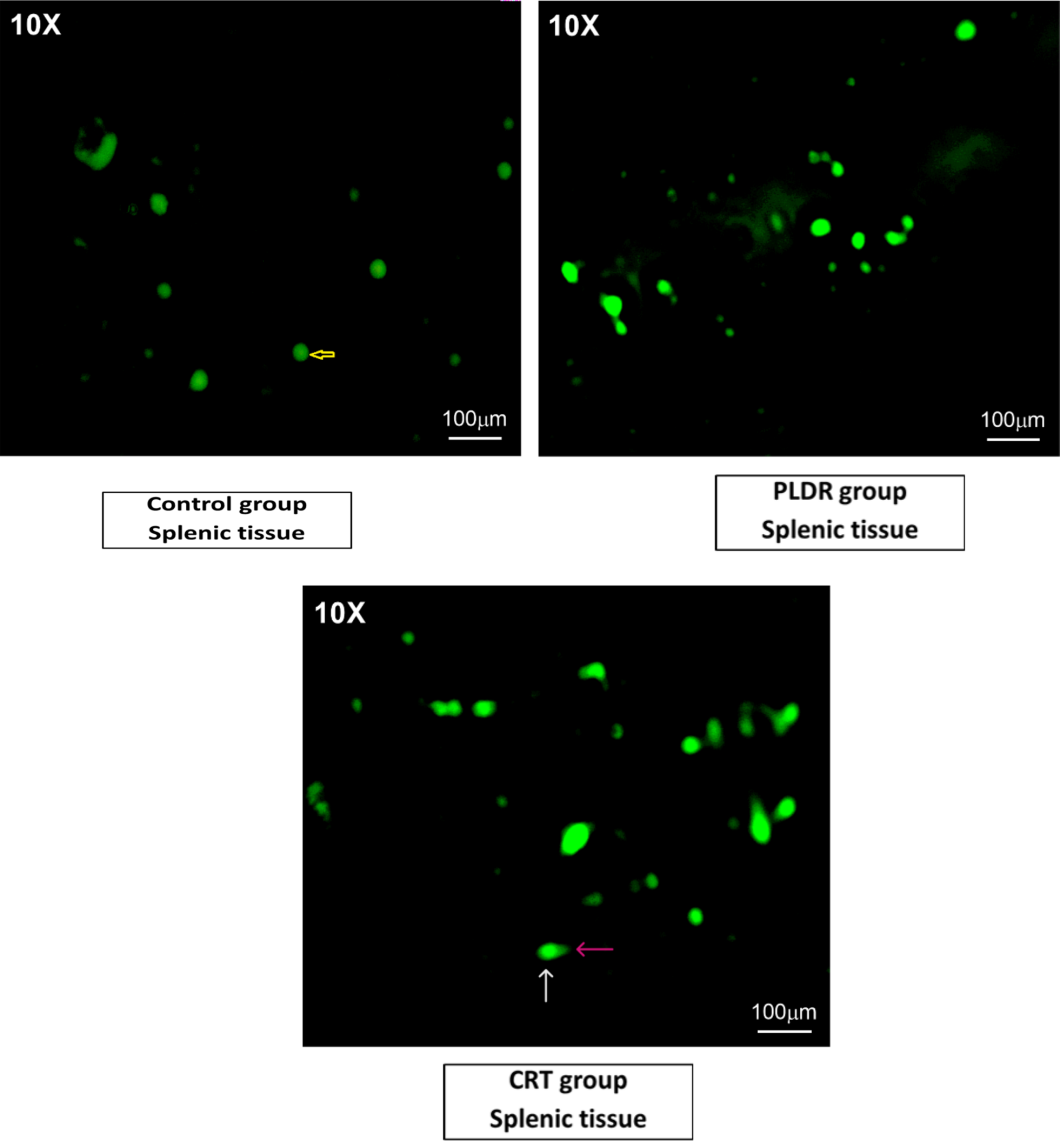
Comet assay images: for measuring DNA damage in spleen cells of rats subjected to various approaches of radiation. Control group received no radiation, CRT group received single 8 Gy and PLDR received pulsed 8 Gy (0.2 Gy × 40fractions with interval 3 min). The images were taken with the LABOMED Fluorescence Microscope LX400.;(catalogue number 9126000, USA). The image scale bar is100µm.Yellow arrow indicates normal cell; white arrow indicates head of comet and pink arrow indicates tail of comet

DNA damage was measured in splenic tissues using the alkaline comet assay method (Fig. 4). As a result of the lysing step using solutions after embedding in the agarose, the cells were destroyed, allowing all components of the cells to diffuse into the gel agarose, except DNA (form nucleoids, containing DNA helical loops). The damaged individual strands and fragments of DNA lost their structure and relax. When applying the electric field for electrophoresis, the small loose DNA fragments, negatively charged, were drawn towards the positive anode, while non- damaged helical DNA with a higher weight was too large to migrate to the positive anode. The migration of damaged DNA to the positive pole was proportional to cell damage. Therefore, each damaged cell formed a comet shape image with head and tail under a fluorescence microscope. The head consists of the integral helical DNA and the tail consists of DNA fragments with varying lengths that have migrated away from the nucleus. Images with higher comets, with higher tail length, and with higher fluorescence intensity (which expresses the tail DNA percentage) indicate greater DNA damage.

**Fig. 5:**
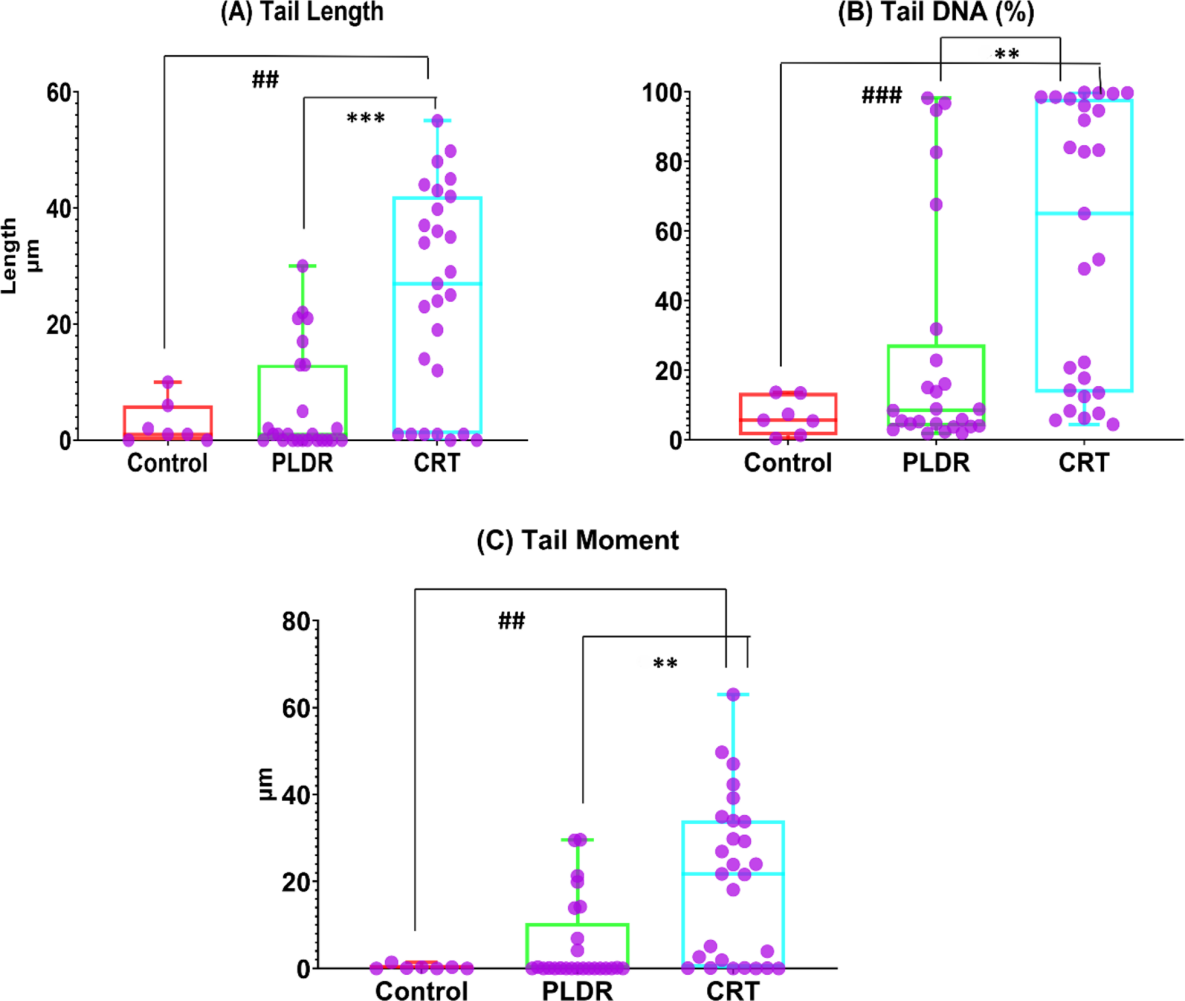
Comet assay parameters **A** Tail length (TL), **B** Tail DNA % (TD%) and **C** Tail Moment (TM) of Splenic tissue. Control group received no radiation, CRT group received single 8 Gy and PLDR received pulsed 8 Gy (0.2 Gy × 40fractions with interval 3 min). This represents the individual data points using the box and whiskers plot. *p ≤ 0.05, **p ≤ 0.005, ***p ≤ 0.0001

The extent of DNA damage was evaluated based on the TL, TD%, and TM = TL × TD. An example of the variations in the mentioned parameters in the samples of the three groups is displayed in Fig. 4. As expected, both irradiated groups showed higher DNA damage than the unirradiated control group, noticed with higher comets and higher tail length (Fig. 4).It was also observed that the DNA damage parameters were higher in CRT group compared to PLDR group, where the mean (SEM) of TL, TD% and TM measurements were 25.4(3.4), 56.5(7.6)% and 20.5(3.5) for CRT, 7.3(1.9), 30.0(7.2)% and 5.7(1.8)for PLDR, and 5.1(2.5),17.6(10.9)% and 2.7(2.4) for the control group as shown in Fig. 5. TL, TD%, and TM levels all showed statistically significant differences between the PLDR and CRT groups with P- value 0.000064, 0.0004, 0.00017, respectively. There were statistically significant differences also between the control and CRT groups within all parameters; TL, TD% and TM (*P* value = 0.0041,0.00060 and 0.0024, respectively). On the other hand, the difference in DNA damage parameters between control and pulsed group was not statistically significant as depicted from the *P* value being > 0.05.

### Immunohistochemistry

#### Histopathological changes in bone marrow and intestinal tissue

**Fig. 6:**
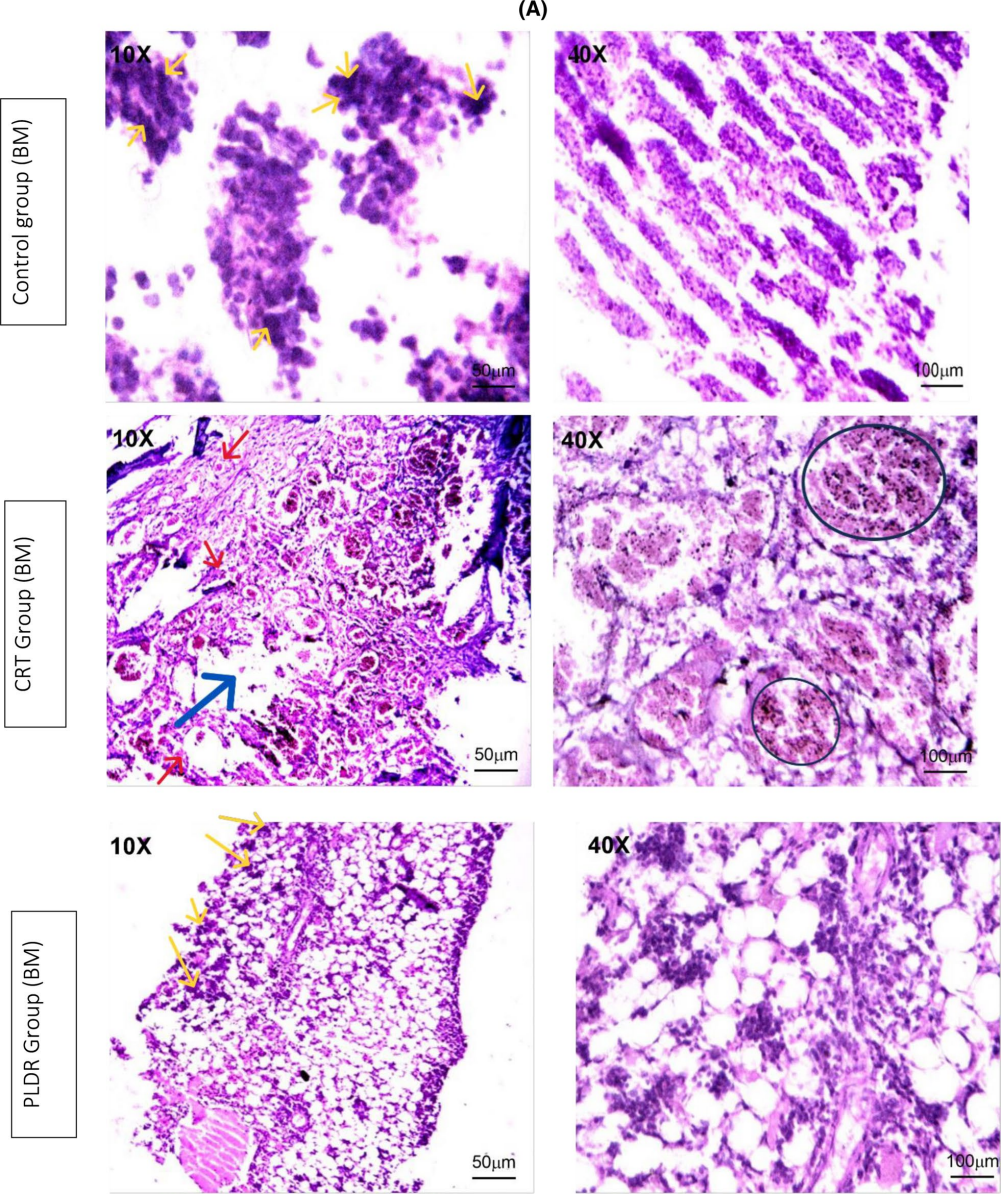

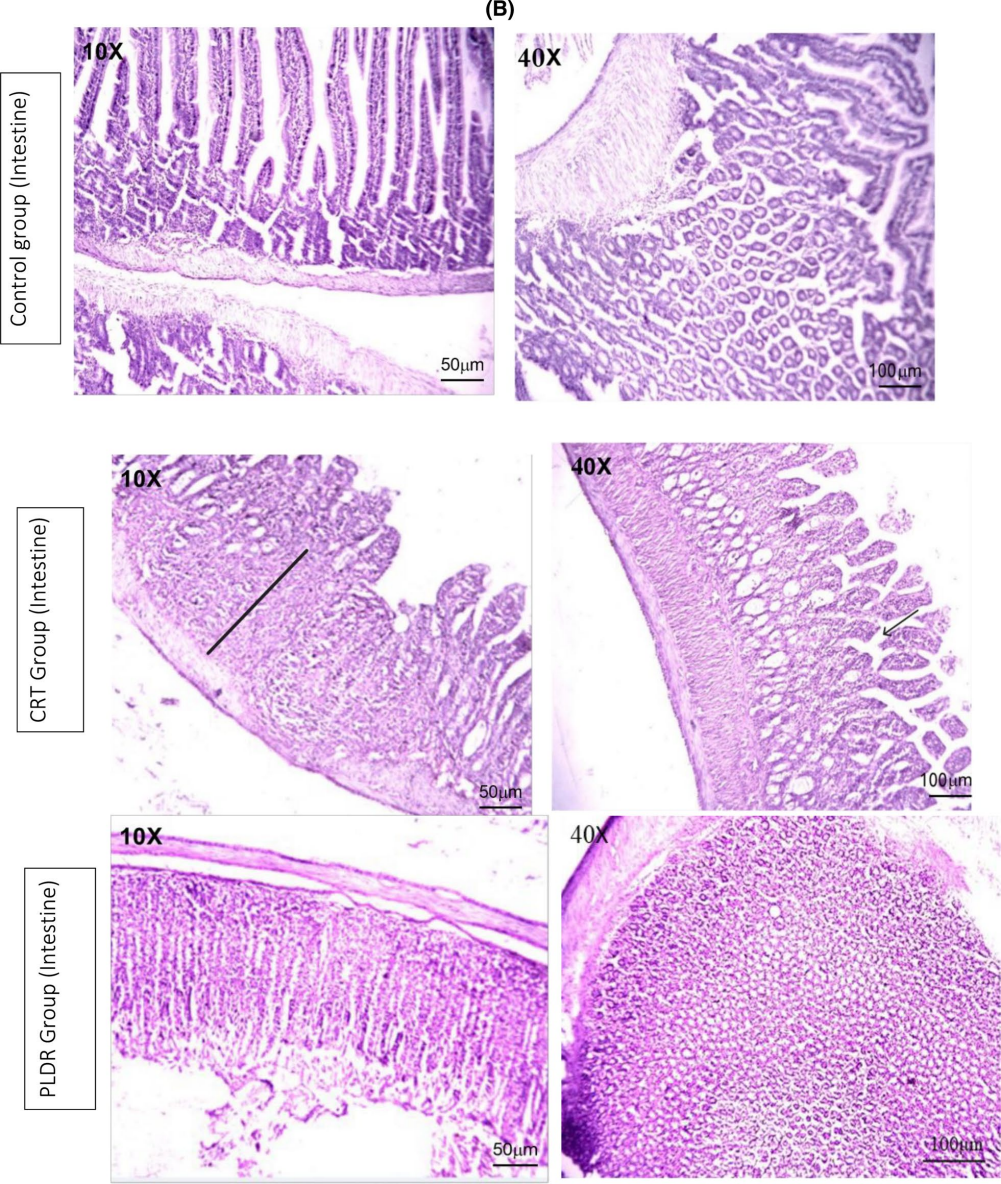
Histopathology images of bone marrow sections (**A**) and intestinal sections (**B**) stained with Hematoxylin and eosin, showing histomorphology features for both PLDR and CRT in comparison to normal tissues. Both sections of bone marrow and intestinal tissue showed a reduced level of toxicity in rats exposed to pulsed radiation (PLDR) than those exposed to equivalent doses of conventional radiation (CRT). Black arrows indicate fibrosis in intestinal tissue, Black straight line indicates atypia, blue arrows indicate fibrosis in BM tissues, yellow arrows indicate cellularity, red arrows indicate extravasation and black circles indicate wide trabecula in BM tissues. The images were taken with a LABOMED Fluorescence Microscope LX400, with the (catalogue number 9126000, USA), Scale bars are shown on images

Bone marrow and intestinal sections of rats from the control group” stained with H&E, showed normal histomorphological features (Fig. 6). Marked histological features of BM atrophy were observed in the CRT group as indicated by the moderate widening of BM trabeculae, deposition of fibrous tissue as well as extravasation and a marked reduction in BM cellularity (Fig. 6A). The PLDR group showed histomorphological features of reduced toxicity in BM tissues when compared to the CRT group irradiated with the same dose (Fig. 6A). Also, an acute histological feature of colitis was observed in intestinal tissues from the CRT group as noticed by a moderate marked Atypia (nuclear enlargement and malorientation) in epithelium (Fig. 6B) associated with degenerative changes, tissue edema, eosinophilic infiltration, and a moderate grade of fibrosis in lamina propria in the intestinal tissue of the CRT group, on the other side, it showed mild degeneration in intestinal tissues of the PLDR group. Histopathology images are shown in (Fig. 6) with magnification power10X and 40X. The images were taken, with the LABOMED Fluorescence Microscope LX400(catalogue number 9126000, USA), images scale bars are shown on figures.

#### IHC staining with TGF-β Changes in Bone Marrow & intestinal tissue

**Fig. 7:**
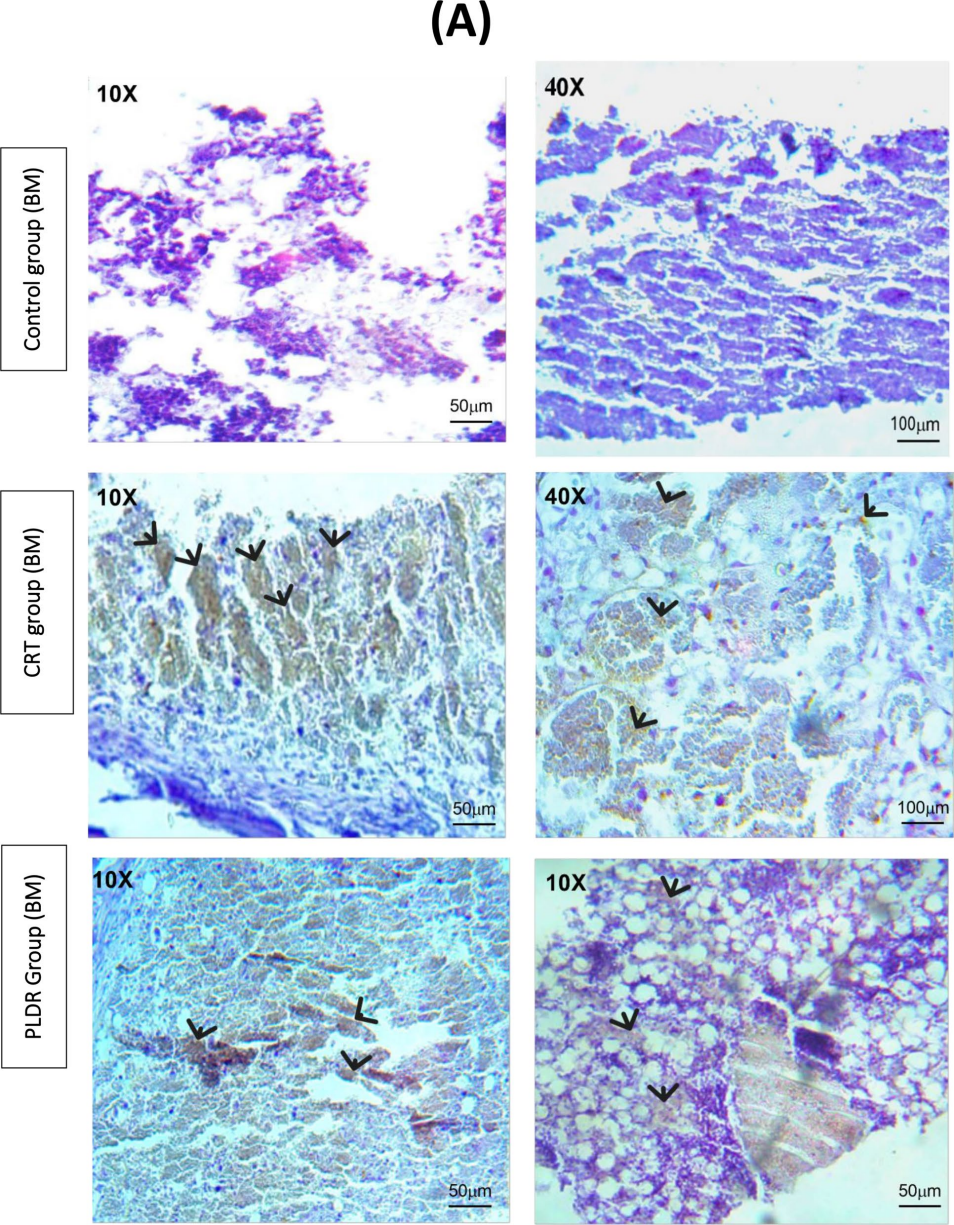

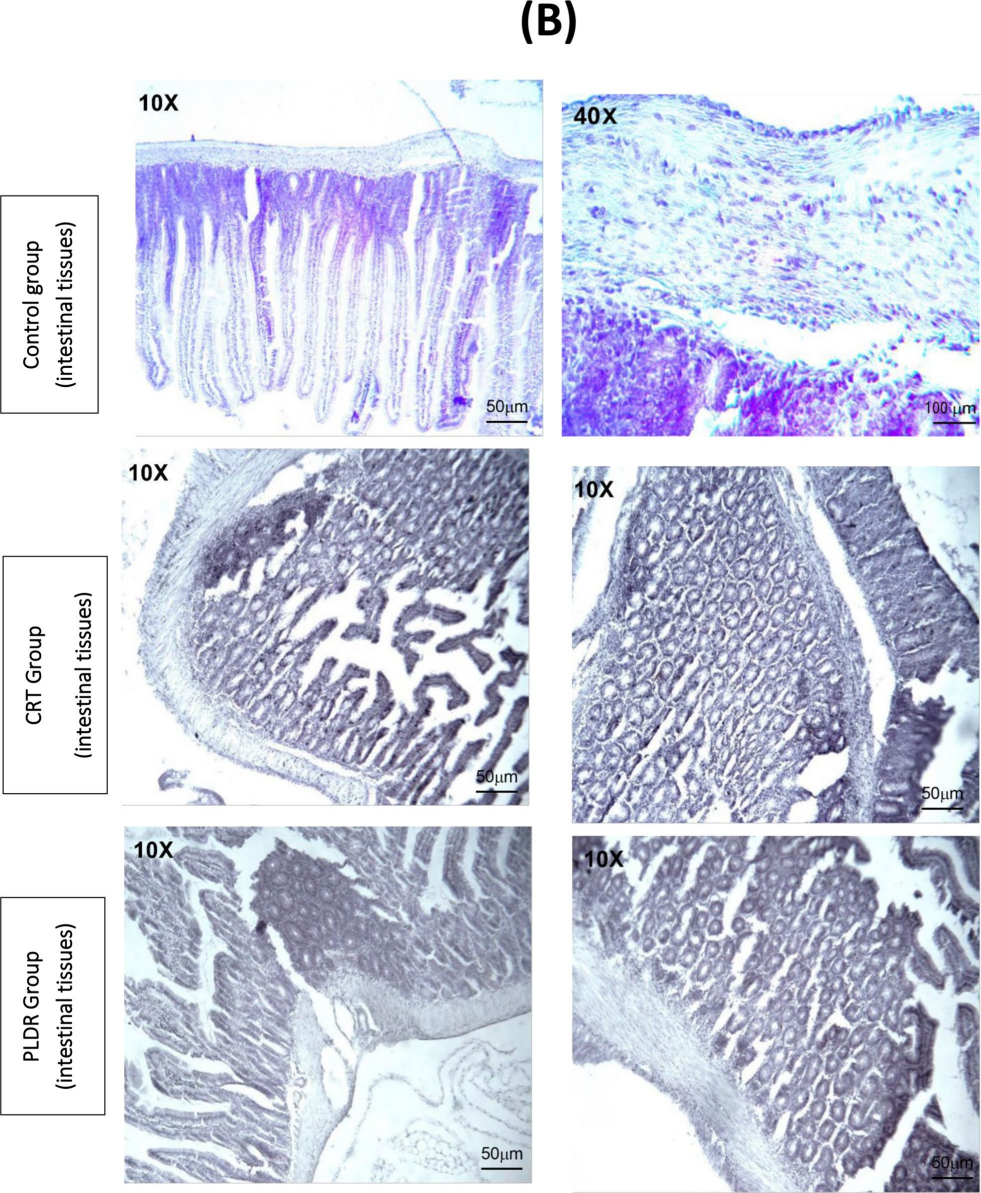
Bone marrow sections (**A**) and intestinal tissue sections (**B**) of rats are stained with Rat specific anti-TGF-β antibody. Figure 7 (A) showed a high brown reaction of TGF-β (arrows)in BM tissues following CRT treatment than those follow PLDR at doses of 8 Gy. In Fig. 7 (B), the CRT images of intestine tissues showed a higher intensity of the brown color than PLDR images, which indicates higher expression for TGF-β. The images were taken with a LABOMED Fluorescence Microscope LX400, with the catalogue number 9126000, USA. TGF-β expression was quantified using the immuno reactive scoring system (IRS), shown in (Fig. 8)

**Fig. 8:**
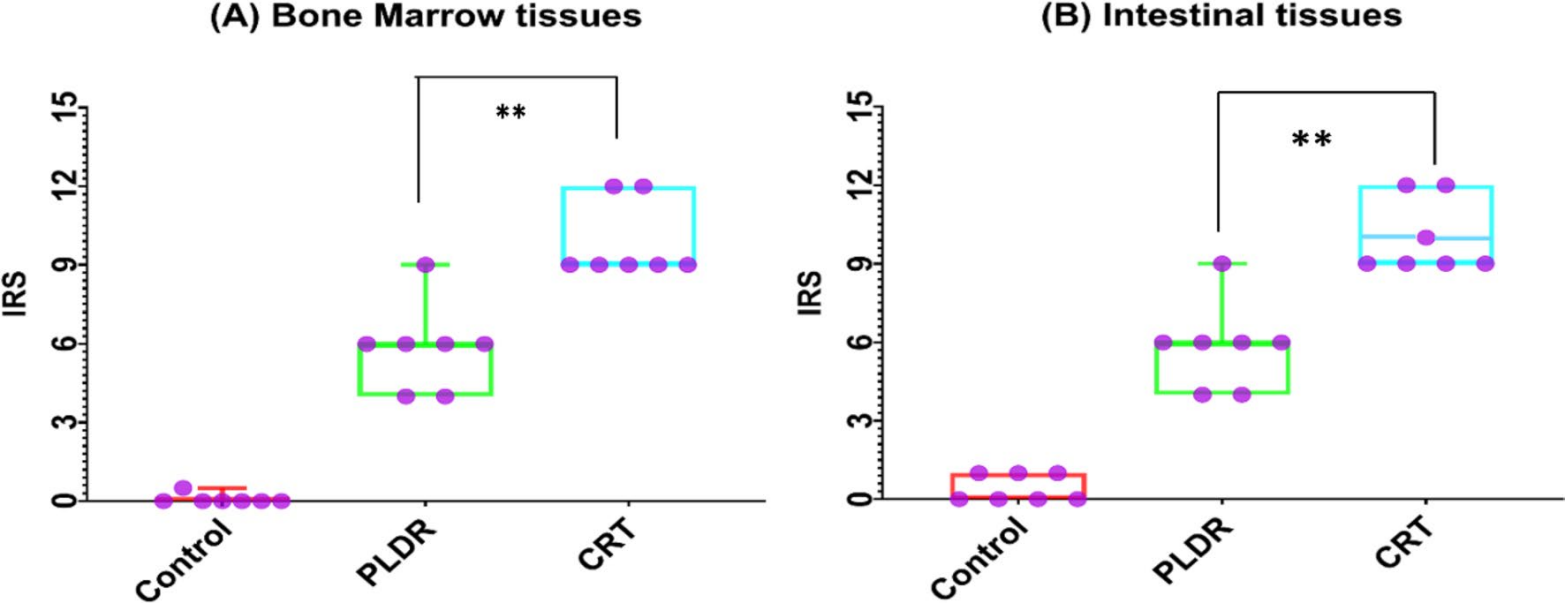
Calculated IRS for negative control group, PLDR group and CRT group of (A) BM tissue and (B) intestinal tissue. Control group received no radiation, CRT group received single 8 Gy and PLDR received Pulsed 8 Gy (0.2 Gy × 40fr with interval 3 min). This graph represents the individual data points using the box and whiskers plot. **p ≤ 0.005

Figure 7 represents assessment of TGF-β expression in bone marrow and intestinal tissues following total body irradiation with 8 Gy. Immunohistochemistry involves the process of quantifying proteins (antigens) in tissues and cells show where a specific given protein is located, depending on antigen -antibodies binding principle. This results in images with colours (blue and brown) which indicate the interaction degree (the details are in the methodology sections). The colours in immunohistochemistry indicate high protein expression (dark brown) and no protein expression (blue), depending on the intensity of the colour. Immunohistochemistry of both BM and intestinal tissues for CRT shows intense positive brown reaction, while for PLDR, shows moderate positive brown reaction (Fig. 7).The immunoreactive score (IRS) defined as multiplication of positive cells percentage by staining intensity and used to interpret the IHC results is shown in Fig. 8.The nonirradiated tissues of the control group exhibited very low protein expression with an average immunoreactive score (IRS) = 0.5(0.2) and 0.07(0.071) for intestinal and BM tissues, respectively. Total body irradiation with both delivery methods induced release of TGF-β in the above tissues. The stain pattern in irradiated tissues in CRT Immunohistochemistry showed higher positive cells and expression of TGF-β than PLDR. The average IRS 10(0.53) and 9.8(0.55), indicates strongly positive reaction for CRT in intestinal and BM tissues, respectively. While the average 5.8(0.63) indicates moderate reaction for PLDR in both BM and intestine tissues (Fig. 8). The immunoreactive scores of both BM and intestinal tissues were significantly different between CRT and PLDR groups. *P* value = 0.0030 and 0.0024, respectively.

## Discussion

Organs and cells with high sensitivity to radiation injury are the hematopoietic system, the gastrointestinal (GI) tract, the brain, spermatogenic cells, and the vascular system [46]. The intestine and bone marrow (BM) are known to be highly sensitive to the acute toxic effects of radiotherapy, both in experimental animal models and in patients subjected to radiotherapy [46]. Gastritis, hemorrhage, nausea, and vomiting are common side effects of whole-body irradiation [46]. This explains choosing BM and intestine in our study and quantifying the extent of the TGF-β expression in their tissues [47]. The spleen as a hemopoietic organ plays a very important role in the body immunity and is known to serve as a reservoir for platelets, lymphocytes, and potentially other cells [48]. Therefore, it was also examined in our work. The study done of Ma et al. [31] examined the effect of radiation on the spleen. They showed that using hematoxylin and eosin caused spleen atrophy following irradiation. In our study, we wanted to detect the damage in spleen using a different approach focusing on genotoxicity, so comet assay was done. A previous comet assay study on spleen lymphocytes showed that very low dose-rate irradiation resulted in a statistically significant increase in nucleoid relaxation (DNA breaks), starting from a dose of 20 cGy [49].

Severe acute toxicity is a crucial factor to consider when planning for reirradiation of recurrent cancers. It was demonstrated in literature that normal cells could be more resistant to radiation with PLDR while preserving the same tumor control as conventional treatment options [5, 7, 31–35]. The PLDR effect on tumor depends on the low dose hyper radiosensitivity. The normal cells’ sparing relies on the low dose rate. The low dose per pulse and periodical beam off intervals between them can enhance the normal tissues' ability to repair sublethal damage and, therefore, reduce toxicity. Several studies investigated the effective dose rate that should be used to give the optimal clinical outcome. Santos et al. tested effective dose rates ranging from 0.083 Gy/min to 1.5 Gy/min by delivering treatment pulses separated by periodical beam off gaps in the order of 10 s, 20 s, 1 min and 3 min. His work did not show significant variations in the survival fractions of human cell lines A549 and PC3 [29]. On the contrary, Terashima et al. used clonogenic experiments to investigate the survival rates of A549 and V79 cancer cells as a function of treatment dose rate and time interval and found that with short beam off interval (10 s, 1 min and 3 min), both cell lines showed significantly reduced survival rates [36]. Ma et al. [30] showed stronger cytotoxic effects at the lower dose rate 0.0677 Gy/min which also favors the use of 3-min interval gaps. Wen et al. used colony assay formation to investigate the time interval in PLDR in terms of the sublethal repair half-time of some normal tissues in the abdominal and pelvic areas. The calculated time interval in the PLDR technique was found to be 2.5 to 3.5 min, and this time interval is equal to the required half life time for repairing these tissues, depending on their type [50]. However as was stated by Ma et al. [9], a 3-minutes interval can be more practical to keep the overall therapy session within 30 min. Hence, in the current work based on the aforementioned discussions, we chose the 3-min interval gap.

Rats prior irradiation were healthy feeding and the increase in weight could be a sign of good digestion and normal growth. Our results showed a continuous decrease in body weight with conventional treatment compared to a slightly increasing weight with PLDR, also, a higher survival rate for PLDR compared to CRT. This agrees with the results of Zhang et al. [31], however our data showed 3 times higher survival rates compared to their results. Many factors could contribute to the disparity between our survival rates and their outcomes, including the variance in the experimental animals used in both studies. In their study, nude mice were used with an average weight in the order of 20–26 gm, while we used adult male Wistar rats with an average weight in the order of 130–150 gm. Yang et al. studied the pathophysiological responses in rat and mouse models of radiation-induced brain injury. They pointed out that mice yield relatively low survival rates after anesthesia and irradiation, and they ascribed that to the small size of the mouse brain which could limit the radiation volume and dose distribution [51]. Takahashi et al. [52] showed the alveolar macrophages decreased much more in mice than in rats after external gamma irradiation. Alveolar macrophages are essential for tissue homeostasis, host defense, clearance of surfactant and cell debris, pathogen recognition, initiation and resolution of lung inflammation, and repair of damaged tissue. While this may offer a potential explanation, we cannot definitively determine that the observed variation between rats and mice in the aforementioned studies accounts for the difference in survival in rats. This might warrant more dedicated investigations.

The comet assay (TL, TD% and TM) which we used to evaluate the DNA damage [49, 53–55] was not considered in many of the PLDR studies. Our results showed lower DNA damage with PLDR and indicated a better DNA sparing compared to conventional treatments. Measurement of the TGF-β has also reinforced that PLDR radiation is qualitatively superior to conventional radiotherapy in terms of the normal tissue sparing. TGF-β is considered to be the master cytokine involved in the fibrotic response in normal tissues exposed to irradiation [39]. TGF-β cellular expression after irradiation showed higher expression in conventional treatment than PLDR and our results are consistent with the study published by Meyer et al. [35].

Future research direction could include the use of tumor bearing animals to investigate the potential gain with PLDR. Benefits will be judged based on the ability of achieving same tumor control with less normal tissue complications and a reduction in toxicity as compared to conventional treatment techniques.

## Conclusion

These data support and promote that there is an obvious difference between PLDR and CRT techniques in terms of toxicity. This opens a window for more possible and various applications of radiotherapy in locally recurrent cases to increase tolerable doses in reirradiation.

## Supplementary Information

Additional file 1.

## Data Availability

The data used and analyzed during the current study are available from the corresponding author on reasonable request.

## Abbreviations

ATM: Ataxia-telangiectasia mutated
BM: Bone marrow
CA: Comet assay
CRT: Conventional radiotherapy
H&E: Haematoxylin and Eosin
HRS: Hyper-radiosensitivity
IHC: Immuno histochemistry
IRR: Increased radio resistance
IRS: Immuno reactive score
MRI: Magnetic resonance imaging
PET: Positron emission tomography
PLDR: Pulsed low dose rate
ROI: Region of interest
SCGE: Single cell gel electrophoresis
TD%: Tail DNA %
TGF-β: Transforming growth factor–β
TL: Tail length
TM: Tail moment

## References

[1] Richards GM, Tomé WA, Robins HI, Stewart JA, Welsh JS, Mahler PA, et al. Pulsed reduced dose-rate radiotherapy: a novel locoregional retreatment strategy for breast cancer recurrence in the previously irradiated chest wall, axilla, or supraclavicular region. Breast Cancer Res Treat. 2009;114(2):307–13. 18389365 10.1007/s10549-008-9995-3

[2] Adkison JB, Tomé W, Seo S, Richards GM, Robins HI, Rassmussen K, et al. Reirradiation of large-volume recurrent glioma with pulsed reduced-dose-rate radiotherapy. Int J Radiat Oncol Biol Phys. 2011;79(3):835–41. 20472350 10.1016/j.ijrobp.2009.11.058

[3] Ma CM, Lin MH, Dai XF, Koren S, Klayton T, Wang L, et al. Investigation of pulsed low dose rate radiotherapy using dynamic arc delivery techniques. Phys Med Biol. 2012;57(14):4613–26. 22750648 10.1088/0031-9155/57/14/4613

[4] Lin MH, Price RA, Li J, Kang S, Li J, Ma CM. Investigation of pulsed IMRT and VMAT for re-irradiation treatments: dosimetric and delivery feasibilities. Phys Med Biol. 2013;58(22):8179–96. 24200917 10.1088/0031-9155/58/22/8179

[5] Wang B, Ren J, Zhang P, Cvetkovic D, Chen X, Chen L, et al. An in-vivo study on pulsed low-dose-rate radiotherapy for prostate cancer. Mathews J Cancer Sci. 2019;4(2):1–7.

[6] Hall EJ, Giaccia AJ. Radiobiology for the Radiologist. Lippincott Williams & Wilkins; 2006. 572 p.

[7] Tomé WA, Howard SP. On the possible increase in local tumour control probability for gliomas exhibiting low dose hyper-radiosensitivity using a pulsed schedule. Br J Radiol. 2007;80(949):32–7. 16945935 10.1259/bjr/15764945

[8] Ma CMC, Luxton G, Orton CG. Point/counterpoint: pulsed reduced dose rate radiation therapy is likely to become the treatment modality of choice for recurrent cancers. Med Phys. 2011;38(9):4909–11. 21978035 10.1118/1.3583794

[9] Ma CMC. Pulsed low dose-rate radiotherapy: radiobiology and dosimetry. Phys Med Biol. 2022;67(3):0301. 10.1088/1361-6560/ac4c2f 35038688

[10] Joiner MC, Marples B, Lambin P, Short SC, Turesson I. Low-dose hypersensitivity: current status and possible mechanisms. Int J Radiat Oncol Biol Phys. 2001;49(2):379–89. 11173131 10.1016/s0360-3016(00)01471-1

[11] Marples B, Collis SJ. Low-dose hyper-radiosensitivity: past, present, and future. Int J Radiat Oncol Biol Phys. 2008;70(5):1310–8. 18374221 10.1016/j.ijrobp.2007.11.071

[12] Wykes SM, Piasentin E, Joiner MC, Wilson GD, Marples B. Low-dose hyper-radiosensitivity is not caused by a failure to recognize DNA double-strand breaks. Radiat Res. 2006;165(5):516–24. 16669705 10.1667/RR3553.1

[13] Hall EJ, Brenner DJ. The dose-rate effect revisited: radiobiological considerations of importance in radiotherapy. Int J Radiat Oncol Biol Phys. 1991;21(6):1403–14. 1938548 10.1016/0360-3016(91)90314-t

[14] Dai X, Tao D, Wu H, Cheng J. Low dose hyper-radiosensitivity in human lung cancer cell line A549 and its possible mechanisms. J Huazhong Univ Sci Technol Med Sci Hua Zhong Ke Ji Xue Xue Bao Yi Xue Ying Wen Ban Huazhong Keji Daxue Xuebao Yixue Yingdewen Ban. 2009;29(1):101–6. 10.1007/s11596-009-0122-4 19224174

[15] Singh B, Arrand JE, Joiner MC. Hypersensitive response of normal human lung epithelial cells at low radiation doses. Int J Radiat Biol. 1994;65(4):457–64. 7908933 10.1080/09553009414550531

[16] Short SC, Kelly J, Mayes CR, Woodcock M, Joiner MC. Low-dose hypersensitivity after fractionated low-dose irradiation in vitro. Int J Radiat Biol. 2001;77(6):655–64. 11403705 10.1080/09553000110041326

[17] Harney J, Short SC, Shah N, Joiner M, Saunders MI. Low dose hyper-radiosensitivity in metastatic tumors. Int J Radiat Oncol Biol Phys. 2004;59(4):1190–5. 15234055 10.1016/j.ijrobp.2003.12.029

[18] Sandur SK, Deorukhkar A, Pandey MK, Pabón AM, Shentu S, Guha S, et al. Curcumin modulates the radiosensitivity of colorectal cancer cells by suppressing constitutive and inducible NF-κB activity. Int J Radiat Oncol Biol Phys. 2009;75(2):534–42. 19735878 10.1016/j.ijrobp.2009.06.034 PMC3090721

[19] Martin LM, Marples B, Lynch TH, Hollywood D, Marignol L. Exposure to low dose ionising radiation: molecular and clinical consequences. Cancer Lett. 2014;349(1):98–106. 24983100 10.1016/j.canlet.2013.12.015

[20] Joiner MC, Lambin P, Malaise EP, Robson T, Arrand JE, Skov KA, et al. Hypersensitivity to very-low single radiation doses: its relationship to the adaptive response and induced radioresistance. Mutat Res. 1996;358(2):171–83. 8946022 10.1016/s0027-5107(96)00118-2

[21] Marples B, Joiner MC, Skov KA. The effect of oxygen on low-dose hypersensitivity and increased radioresistance in Chinese hamster V79–379A cells. Radiat Res. 1994;138(1 Suppl):S17-20. 8146317

[22] Xu B, Kim ST, Lim DS, Kastan MB. Two molecularly distinct G2/M checkpoints are induced by ionizing irradiation. Mol Cell Biol. 2002;22(4):1049–59. 11809797 10.1128/MCB.22.4.1049-1059.2002 PMC134638

[23] Short SC, Woodcock M, Marples B, Joiner MC. Effects of cell cycle phase on low-dose hyper-radiosensitivity. Int J Radiat Biol. 2003;79(2):99–105. 12569013

[24] Krempler A, Deckbar D, Jeggo PA, Löbrich M. An imperfect G2M checkpoint contributes to chromosome instability following irradiation of S and G2 phase cells. Cell Cycle Georget Tex. 2007;6(14):1682–6. 10.4161/cc.6.14.4480 17637566

[25] Leonard BE. Thresholds and transitions for activation of cellular radioprotective mechanisms - correlations between HRS/IRR and the “inverse” dose-rate effect. Int J Radiat Biol. 2007;83(7):479–89. 17538798 10.1080/09553000701370902

[26] Mitchell CR, Folkard M, Joiner MC. Effects of exposure to low-dose-rate (60)co gamma rays on human tumor cells in vitro. Radiat Res. 2002;158(3):311–8. 12175308 10.1667/0033-7587(2002)158[0311:eoetld]2.0.co;2

[27] Matsuya Y, McMahon SJ, Tsutsumi K, Sasaki K, Okuyama G, Yoshii Y, et al. Investigation of dose-rate effects and cell-cycle distribution under protracted exposure to ionizing radiation for various dose-rates. Sci Rep. 2018;8(1):8287. 29844494 10.1038/s41598-018-26556-5 PMC5974424

[28] Todorovic V, Prevc A, Zakelj MN, Savarin M, Bucek S, Groselj B, et al. Pulsed low dose-rate irradiation response in isogenic HNSCC cell lines with different radiosensitivity. Radiol Oncol. 2020;54(2):168–79. 32229678 10.2478/raon-2020-0015 PMC7276640

[29] Liu H, Shen C, Klages P, Albuquerque K, Ma CM, Jia X. Investigation of dose-rate effects in pulsed low dose rate radiotherapy. Mathews J Cancer Sci. 2022;7(2):1–6.

[30] Ma C, Mu Z, Tafo AG, Chen L. Variation of cytotoxic effect with pulsed dose sequence and low dose rate radiation. Int J Radiat Oncol. 2010;78(3):S629.

[31] Zhang P, Wang B, Chen X, Cvetkovic D, Chen L, Lang J, et al. Local tumor control and normal tissue toxicity of pulsed low-dose rate radiotherapy for recurrent lung cancer. Dose-Response. 2015;13(2):1559325815588507. 26675811 10.1177/1559325815588507 PMC4674173

[32] Dilworth JT, Krueger SA, Dabjan M, Grills IS, Torma J, Wilson GD, et al. Pulsed low-dose irradiation of orthotopic glioblastoma multiforme (GBM) in a pre-clinical model: effects on vascularization and tumor control. Radiother Oncol J Eur Soc Ther Radiol Oncol. 2013;108(1):149–54. 10.1016/j.radonc.2013.05.022 23791366

[33] Park SS, Chunta JL, Robertson JM, Martinez AA, Oliver Wong CY, Amin M, et al. MicroPET/CT imaging of an orthotopic model of human glioblastoma multiforme and evaluation of pulsed low-dose irradiation. Int J Radiat Oncol. 2011;80(3):885–92. 10.1016/j.ijrobp.2011.01.045 21489704

[34] Meyer K, Krueger SA, Kane JL, Wilson TG, Hanna A, Dabjan M, et al. Pulsed radiation therapy with concurrent cisplatin results in superior tumor growth delay in a head and neck squamous cell carcinoma murine model. Int J Radiat Oncol Biol Phys. 2016;96(1):161–9. 27511853 10.1016/j.ijrobp.2016.04.031

[35] Meyer JE, Finnberg NK, Chen L, Cvetkovic D, Wang B, Zhou L, et al. Tissue TGF-β expression following conventional radiotherapy and pulsed low-dose-rate radiation. Cell Cycle. 2017;16(12):1171–4. 28486014 10.1080/15384101.2017.1317418 PMC5499842

[36] Terashima S, Hosokawa Y, Tsuruga E, Mariya Y, Nakamura T. Impact of time interval and dose rate on cell survival following low-dose fractionated exposures. J Radiat Res (Tokyo). 2017;58(6):782–90. 28595296 10.1093/jrr/rrx025 PMC5710595

[37] Joiner M, Kogel A van der, editors. Basic clinical radiobiology. 4th ed. London: Hodder Arnold; 2009. 375 p.

[38] Pelton RW, Moses HL. The beta-type transforming growth factor. Mediators of cell regulation in the lung. Am Rev Respir Dis. 1990;142(6 Pt 2):S31-35. 2174660 10.1164/ajrccm/142.6_Pt_2.S31

[39] Rübe CE, Uthe D, Schmid KW, Richter KD, Wessel J, Schuck A, et al. Dose-dependent induction of transforming growth factor β (TGF-β) in the lung tissue of fibrosis-prone mice after thoracic irradiation. Int J Radiat Oncol Biol Phys. 2000;47(4):1033–42. 10863076 10.1016/s0360-3016(00)00482-x

[40] Singh NP, McCoy MT, Tice RR, Schneider EL. A simple technique for quantitation of low levels of DNA damage in individual cells. Exp Cell Res. 1988;175(1):184–91. 3345800 10.1016/0014-4827(88)90265-0

[41] Tice RR, Andrews PW, Singh NP. The single cell gel assay: a sensitive technique for evaluating intercellular differences in DNA damage and repair. Basic Life Sci. 1990;53:291–301. 2282039 10.1007/978-1-4613-0637-5_23

[42] Czarnek K, Siwicki AK. Genotoxicity of chromium (III) and cobalt (II) and interactions between them. Curr Issues Pharm Med Sci. 2021;34(3):142–8.

[43] STA-355-comet-assay-kit manual.pdf.

[44] De Boeck M, Touil N, De Visscher G, Vande PA, Kirsch-Volders M. Validation and implementation of an internal standard in comet assay analysis. Mutat Res. 2000;469(2):181–97. 10984679 10.1016/s1383-5718(00)00075-9

[45] Fedchenko N, Reifenrath J. Different approaches for interpretation and reporting of immunohistochemistry analysis results in the bone tissue - a review. Diagn Pathol. 2014;29(9):221. 10.1186/s13000-014-0221-9 PMC4260254 25432701

[46] Kiang JG, Olabisi AO. Radiation: a poly-traumatic hit leading to multi-organ injury. Cell Biosci. 2019;9(1):25. 30911370 10.1186/s13578-019-0286-y PMC6417034

[47] Farhood B, Khodamoradi E, Hoseini-Ghahfarokhi M, Motevaseli E, Mirtavoos-Mahyari H, Eleojo Musa A, et al. TGF-β in radiotherapy: Mechanisms of tumor resistance and normal tissues injury. Pharmacol Res. 2020;155: 104745. 32145401 10.1016/j.phrs.2020.104745

[48] Chin AL, Aggarwal S, Pradhan P, Bush K, Von Eyben R, Koong AC, et al. The role of bone marrow and spleen irradiation in the development of acute hematologic toxicity during chemoradiation for esophageal cancer. Adv Radiat Oncol. 2018;3(3):297–304. 30202799 10.1016/j.adro.2018.02.005 PMC6128098

[49] Osipov AN, Klokov DY, Elakov AL, Rozanova OM, Zaichkina SI, Aptikaeva GF, et al. Comparison in vivo Study of Genotoxic Action of High- Versus Very Low Dose-Rate gamma-Irradiation. Nonlinearity Biol Toxicol Med. 2004;2(3):223–32. 19330145 10.1080/15401420490507521 PMC2657484

[50] Wen X, Qiu H, Shao Z, Liu G, Liu N, Chen A, et al. Pulsed low-dose rate radiotherapy has an improved therapeutic effect on abdominal and pelvic malignancies. J Zhejiang Univ-Sci B. 2021;22(9):774–81. 34514757 10.1631/jzus.B2000793 PMC8435343

[51] Yang L, Yang J, Li G, Li Y, Wu R, Cheng J, et al. Pathophysiological responses in rat and mouse models of radiation-induced brain injury. Mol Neurobiol. 2017;54(2):1022–32. 26797684 10.1007/s12035-015-9628-x PMC5310567

[52] Takahashi S, Kubota Y, Sato H. Difference between C3H mice and Wistar rats in the effect of external gamma-irradiation on 59Fe release from alveolar macrophage-ingested 59Fe-iron hydroxide colloid. J Radiat Res (Tokyo). 1991;32(3):262–6. 1791589 10.1269/jrr.32.262

[53] Surniyantoro HNE, Darlina TR. Alkaline comet assay as a predictor of DNA damage in medical radiation workers. J Phys Conf Ser. 2020;1436(1):012023.

[54] El-Marakby SM, Abdelgawad MH, Awd MM, Eraba KM, Desouky OS. DNA damage detection after chronic exposure and radio-adaptive response of naturally occurring radioactive materials (NORM). Arab J Nucl Sci Appl. 2021;54(3):34–45.

[55] Mondal T, Nautiyal A, Patwari A, Ozukum A, Mitra D, Goel A, et al. DNA double strand breaks, repair and apoptosis following 511 keV γ-rays exposure using 18 Fluorine positron emitter: an in-vitro study. Biomed Phys Eng Express. 2018;2:4.

